# Web-Based Dynamic Nomograms for Predicting Overall Survival and Cancer-Specific Survival in Breast Cancer Patients with Lung Metastases

**DOI:** 10.3390/jpm13010043

**Published:** 2022-12-26

**Authors:** Kangtao Wang, Yuqiang Li, Dan Wang, Zhongyi Zhou

**Affiliations:** 1 Department of General Surgery, Xiangya Hospital, Central South University, Changsha 410008, China; 2 National Clinical Research Center for Geriatric Disorders, Xiangya Hospital, Central South University, Changsha 410008, China

**Keywords:** breast cancer lung metastasis (BCLM), estrogen receptor (ER), web-based dynamic nomogram, SEER database, overall survival (OS)

## Abstract

Background: 60–70% of patients who die from breast cancer have lung metastases. However, there is a lack of readily available tools for accurate risk stratification in patients with breast cancer lung metastases (BCLM). Therefore, a web-based dynamic nomogram was developed for BCLM to quickly, accurately, and intuitively assess overall and cancer-specific survival rates. Methods: Patients diagnosed with BCLM between 2004 and 2016 were extracted from the Surveillance, Epidemiology, and Final Results (SEER) database. After excluding incomplete data, all patients were randomly assigned to training and validation cohorts (2:1). Patients’ basic clinical information, detailed pathological staging and treatment information, and sociological information were included in further analysis. Nomograms were constructed following the evaluations of the Cox regression model and verified using the concordance index (C-index), calibration curves, time-dependent receiver operating characteristic (ROC) curves, and decision curve analysis (DCA). Web-based dynamic nomograms were published online. Results: 3916 breast cancer patients with lung metastases were identified from the SEER database. Based on multivariate Cox regression analysis, overall survival (OS) and cancer-specific survival (CSS) are significantly correlated with 13 variables: age, marital status, race, grade, T stage, surgery, chemotherapy, bone metastatic, brain metastatic, liver metastatic, estrogen receptor (ER), progesterone receptor (PR), and human epidermal growth factor receptor-2 (HER2). These are included in the construction of the nomogram of OS and CSS. The time-dependent receiver operating characteristic curve, decision curve analysis, consistency index, and calibration curve prove the distinct advantages of the nomogram. Conclusions: Our web-based dynamic nomogram effectively integrates patient molecular subtype and sociodemographic characteristics with clinical characteristics and guidance and can be easily used. ER-Negative should receive attention in diagnosing and treating BCLM.

## 1. Background

Breast cancer is the most diagnosed cancer and the second leading cause of cancer-related deaths among women in developed countries [1]. More than 90% of deaths caused by breast cancer are due to metastasis-related complications [2]. However, metastasis is a little-known process that begins when tumor cells are separated from the primary tumor and injected into the bloodstream [3]. These circulating tumor cells (CTCs) eventually stay in the capillary bed of distant organs and extravasate into the parenchyma through the blood vessel wall, resulting in metastatic colonies in secondary sites [4]. The bone, brain, liver, and lungs are more likely to metastasize by breast cancer, knowns as organ orientation [5]. For breast cancer patients with metastases, 30–60% of patients have lesions in the bones, 4–10% in the brain, 15–32% in the liver, 21–32% in the lungs, and in many reported cases—multiple simultaneous transfers [6].

Breast cancer lung metastases (BCLM) often occurs within five years of the initial breast cancer diagnosis and significantly impacts the morbidity and mortality of patients [7]. Physiologically, these metastases can disrupt normal lung function, leading to coughing, breathing difficulties, hemoptysis, and death. The prognosis for patients with metastases confined to the lungs is inferior, with a median survival of only 25 months [8]. It is estimated that 60–70% of patients who die of breast cancer have lung metastases [9]. Therefore, accurate risk stratification for identifying BCLM is essential for treatment selection and prognostic evaluation. In addition, many clinicopathological factors, especially the molecular subtypes of breast cancer, could affect the occurrence and prognosis of BC lung metastasis [10]. ER-positive and PR-positive patients are more likely to develop bone metastases, while patients with more aggressive subtypes such as HER-2-positive and triple-negative tumors are more likely to develop visceral and brain metastases [11]. Moreover, the prognostic value of molecular subtypes in breast cancer patients with lung metastases remains controversial [12]. In addition, the influence of sociodemographic factors and clinicopathological characteristics on the incidence and prognosis of newly diagnosed breast cancer lung metastasis is still unclear.

Accurate risk stratification of BCLM patients is essential for treatment selection and prognosis assessment. We intend to explore the risk factors for BCLM through big data, combined with sociodemographic factors and molecular subtypes of breast cancer, to determine the high-risk factors for BCLM patients at the diagnosis. As a visual representation of mathematical models, nomograms can integrate certain features to estimate specific endpoints and provide practical and comprehensive predictions for clinical practice. Therefore, we aim to create a SEER-based prognostic nomogram for BCLM patients and accurately and conveniently assess overall survival (OS) and cancer-specific survival (CSS).

## 2. Materials and Methods

### 2.1. Study Design and Patient Selection

We used the SEER*Stat 8.3.8 program to retrieve and download patient information from the SEER 18 database. The SEER*Stat program is a public country registration database containing cancer incidence data in 18 regions of the United States, accounting for approximately 34.6% of the total population, and detailed patient data.

**Figure 1:**
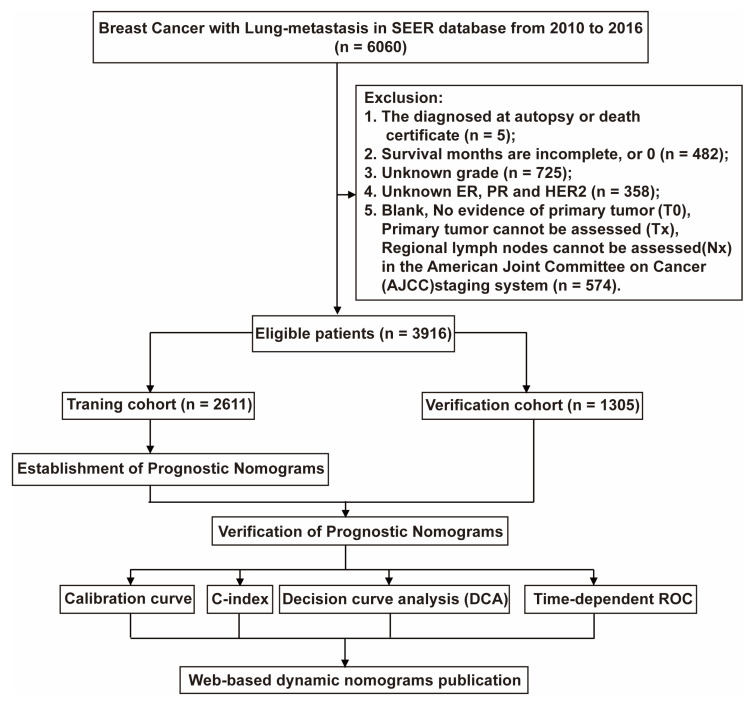
The flowchart of patients was identified in the study. A total of 6060 BCML patients were included from the SEER database, and we excluded 2144 patients based on exclusion criteria. The 3916 patients who met the requirements were randomly divided into training and validation cohort by 2:1. The training cohort was used to construct Norman diagrams, and the validation cohort was used to evaluate the effect of the building model. We validated our constructed Norman plots using the Calibration curve, the C-index decision curve analysis (DAC), and time-dependent ROC methods.

The trial population included adult female breast cancer patients diagnosed with lung metastases from 2010 to 2016. We included the patients’ race, age, marital status, detailed tumor node metastasis (TNM) staging, and whether they had undergone primary site radical resection, chemotherapy, ER, PR, HER2 receptor status, bone metastasis, brain metastasis, or liver metastasis. We excluded patients who had no pathological results and no complete information. Finally, 3916 patients were included in our study, and the selection flow chart is shown in Figure 1. This type of retrospective research does not require an ethics committee review.

### 2.2. Statistical Analysis and Dynamic Nomogram Publication

Patients were randomly divided into 2:1 training and verification groups, 2611 and 1305 cases. Univariate Cox proportional hazards regression analysis was developed to identify independent prognostic factors to construct prognostic factors. Based on univariate analysis results (*p*-value < 0.1), a multivariate Cox proportional hazard regression analysis was performed to construct a nomogram of significant variables (*p*-value < 0.05) in the training group. We use 1-year, 3-year, and 5-year OS and CSS for analysis in the nomogram. The consistency index (C-index) is used to evaluate the discrimination ability and accuracy of the nomogram. The identification and calibration are evaluated by bootstrapping 1000 times. Decision curve analysis (DCA) is used to evaluate the advantages and benefits of our new forecasting model compared with another single factor. In addition, time-dependent ROC is used to evaluate the effectiveness of the OS and CSS of the models we built. After validation, the web-based dynamic nomograms for BCLM OS and CSS are published online through the r package “DynNom” and shinyapps.io (https://www.shinyapps.io/) (accessed on 20 December 2022).

All these statistical methods are packaged using r version 4.2.0 (http://www.r-project.org) (accessed on 20 December 2022). In the two-tailed test, statistical significance is set to *p* < 0.05.

## 3. Results

### 3.1. Patient Characteristics

Our study excluded 2144 cases initially (lack of autopsy or death certificate diagnosis (*n* = 5); survival months are incomplete, or 0 (*n* = 482); lack of histological grade (*n* = 725); lacking ER, PR, and HER2 status (*n* = 358); T0 and Tx according to the 6th edition AJCC staging (*n* = 574) (Figure 1)), and 3916 cases were finally included (2611 cases were training cohort and 1305 cases were Verification cohort). Table 1 summarizes the patient characteristics. Among all BCLM patients, the median age was 62, 41.2% were married, and 72.3% were white. Pathologically, more than half of the patients were poorly differentiated tumors (55.8%), and 42.2% of patients had T4. Only 30.2% of patients underwent surgery in terms of treatment, but most patients underwent chemotherapy (62.7%). Bone metastasis is the most common in patients with BCLM (53.5%), followed by liver metastasis (26.7%). The probability of HER2 negative is the highest, more than 70.7%. In addition, there are 460 triple-negative breast cancer (TNBC) patients, accounting for 11.7%. The longest follow-up of the entire cohort is 82 months, the average OS is 20.9 months, and the CSS is similar to the OS, which is 21.0 months.

### 3.2. Establishment of a Prognostic Nomogram

**Figure 2:**
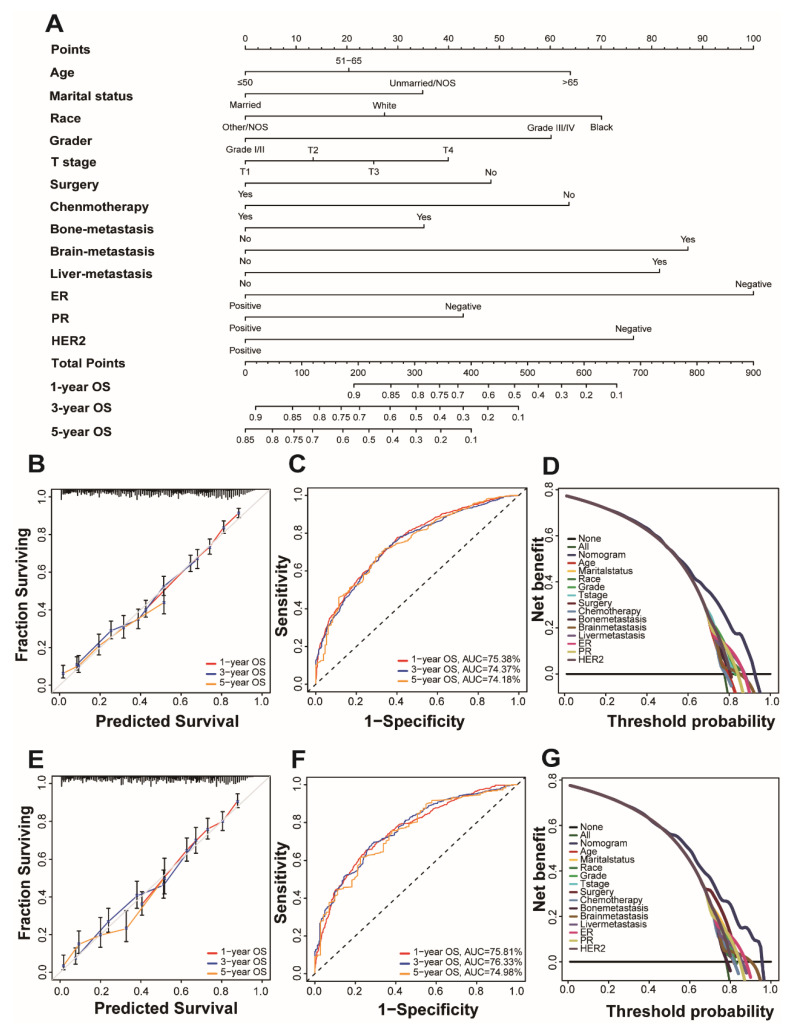
Nomogram and model assessment for predicting 1-, 3-, and 5-year overall survival (OS) in BCLM patients. (**A**) The OS nomogram for BCLM patients. The calibration plots in the (**B**) training and (**E**) validation cohorts for 1-year, 3-year, and 5-year OS. AUC values of ROCs of the nomograms for 1-year, 3-year, and 5-year OS. The values in brackets B and E represent the area under the ROC curves (AUC). (**C**) for the training cohort and (**F**) for the validation cohort. Decision curve analyses (DCA) of the nomogram and other factors’ overall survival. (**D**) for the training cohort and (**G**) for the validation cohort.

**Figure 3:**
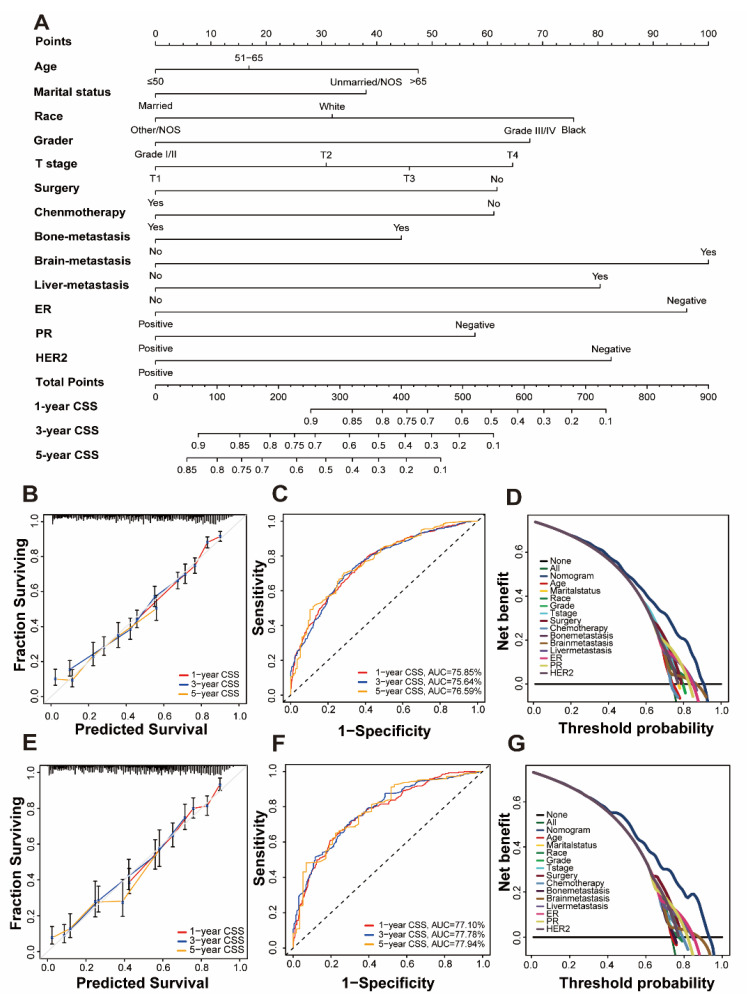
Nomogram and model assessment for predicting 1-, 3-, and 5-year cancer-specific survival (CSS) in BCLM patients. (**A**) The CSS nomogram for BCLM patients. The calibration plots in the (**B**) training and (**E**) validation cohorts for 1-year, 3-year, and 5-year CSS. AUC values of ROCs of the nomograms for 1-year, 3-year, and 5-year OS. The values in brackets B and E represent the area under the ROC curves (AUC). (**C**) for the training cohort and (**F**) for the validation cohort. Decision curve analyses (DCA) of the nomogram and other factors’ overall survival. (**D**) for the training cohort and (**G**) for the validation cohort.

Univariate and multivariate Cox regression analysis is used to calculate the weights of variables in OS and CSS (expressed as OR) and identify independent risk factors. Variables with significant differences in univariate analysis are included in the Cox regression model of multivariate analysis, in which OS and CSS are significantly related to 13 variables, namely age, marital status, race, grade, T stage, surgery, chemotherapy, bone metastatic, brain metastatic, liver metastatic, ER, PR, and HER2. Patient gender, insurance status, lymph node metastasis, and radiation therapy were excluded. (Table 2 and Table 3). All important variables are integrated to build a nomogram of OS and CSS. The nomograms of 1-year, 3-year, and 5-year OS are shown in Figure 2A, and the nomograms of 1-year, 3-year, and 5-year CSS are shown in Figure 3A. After adding up the scores associated with each variable and placing the total score in the bottom tier, the probability of OS and CSS can be estimated at 1, 3, and 5 years.

### 3.3. Verification of Prognostic Nomogram

The concordance index (C-index), calibration curves, time-dependent receiver operating characteristic (ROC) curves, and decision curve analysis (DCA) methods were used to verify the superiority of our nomogram. 

In the C index evaluation, the training group’s OS and CSS C indexes were 0.702 (95% CI, 0.687–0.716) and 0.710 (95% CI, 0.693–0.727). The OS and CSS C indexes of the validation group were, respectively, 0.703 (95% CI, 0.682–0.723), and 0.715 (95% CI, 0.692–0.737) (Table 4).

Furthermore, high reliability was demonstrated by calibration curves in OS (Figure 2B,D) and CSS (Figure 3B,D). Time-dependent ROC curves determine the sensitivity and specificity of predicting the prognosis of BCLM. Figure 2C,F shows the 1-year, 3-year, and 5-year values of the area under the curve of the OS nomogram (training group: 1-year operating system, 75.38%; 3-year OS, 74.37%; and 5-year OS, 74.18%; Verification Group: 1-year OS, 75.81%; 3-years OS, 76.33%; and 5-years OS, 74.98%). The AUC value of the predicted CSS nomogram is shown in Figure 3 (training group: 1-year CSS, 75.85%; 3-year CSS, 75.64%; and 5-year CSS, 76.59%; Verification group: 1-year CSS, 77.10%; 3-year period CSS, 77.78%; and 5-year CSS, 77.97%). In addition, due to the slight deviation from the reference line, Figure 2D,E is for the OS and Figure 3D,E is for CSS. 

DCA can compare nomograms and other factors to help clinicians make beneficial decisions. Figure 2D,G shows the DCA curves of OS for nomogram and other clinical factors, and Figure 3D,G represents CSS. Compared with all other clinical factors, the DCA of the nomogram showed a superior net benefit, suggesting an excellent clinical application of the nomogram in this study.

### 3.4. Risk Stratification and Web-Based Dynamic Nomogram Publication

**Figure 4:**
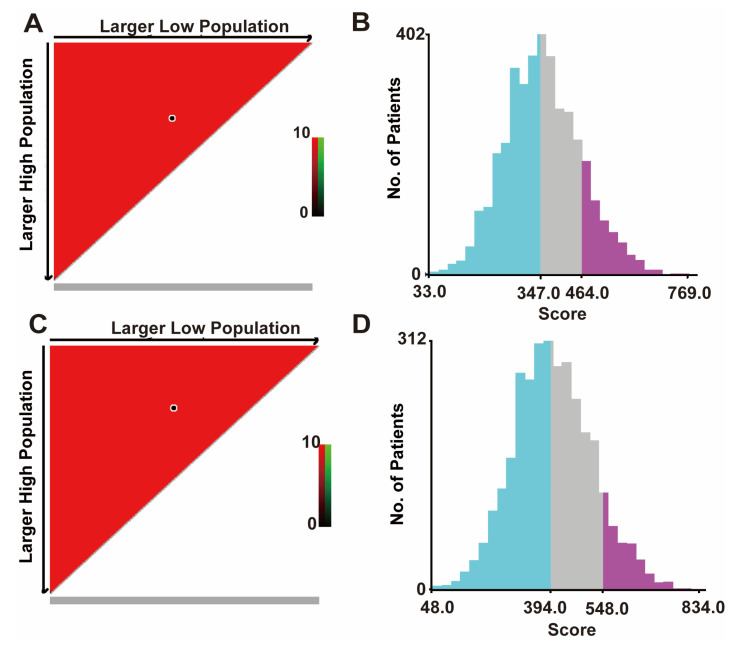
The cut-off values were calculated using X-tile based on the total scores. (**A**) The black dot in the figure indicates the OS distinction point selected by X-tile software. (**B**) The cut-off values of OS are 347 and 464. The blue color is the low-risk group, the gray color is the medium-risk group, and the purple color is the high-risk group. (**C**) The black dots in the figure represent the CSS distinguishing points selected by X-tile software. (**D**) The cut-off values of CSS are 394 and 548. OS, overall survival; the blue color is the low-risk group, the gray color is the medium-risk group, and the purple color is the high-risk group. CSS, cancer-specific survival. All patients under our group price adjustment are under the OS, CSS curve.

X-tile software (version 3.6.1; Yale University, New Haven, CT, USA) was used to calculate the cut-off value by adding the scores associated with each variable to obtain the total score for patients with BCLM. The cut-off values for OS are 347 and 464, and the cut-off values for CSS are 394 and 548 (Figure 4A,B). Therefore, BCLM patients are classified as high-risk (score > 464), medium-risk (347 < score ≤ 464), and low-risk (score ≤ 347) for OS. In addition, BCLM patients were classified as high-risk (score > 548), medium-risk (394 < score ≤ 548), and low-risk (score ≤ 394) for CSS (Figure 4C,D). 

**Figure 5:**
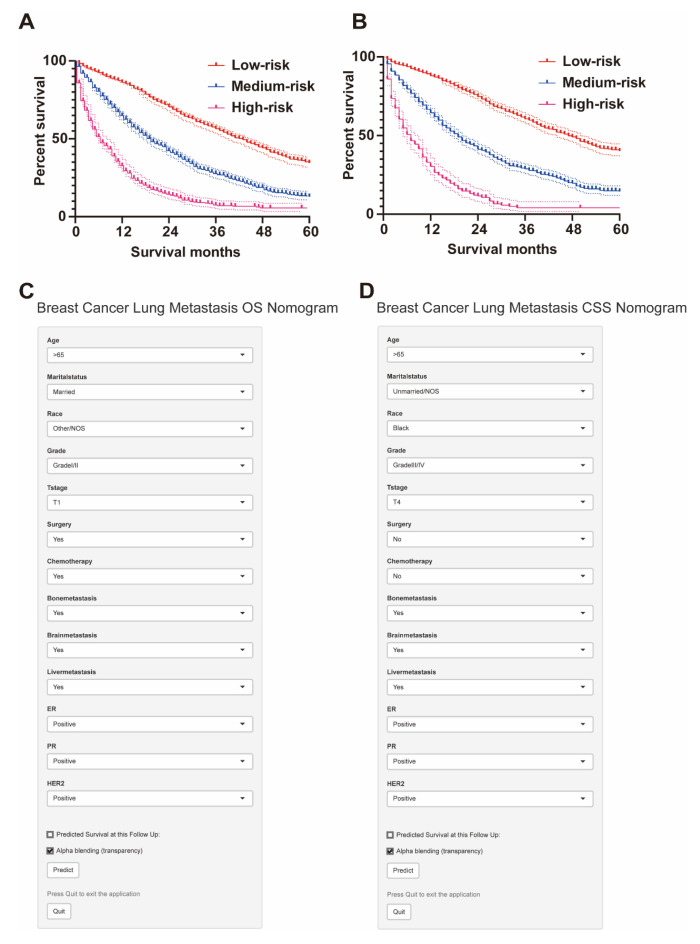
Risk stratification by the total score for subgroup survival analysis and web-based dynamic nomogram. (**A**) The 5-year OS rate is the highest in the low-risk group at 34.5%, followed by the intermediate-risk group at 12.9%, and the worst at 4.9% in the high-risk group. (**B**) The low-risk group had the highest 5-year CSS rate at 40.1%, followed by the intermediate-risk group with 14.5%, and the high-risk group with 4.6%. (**C**) Web-based dynamic nomogram of OS of BCLM patients (https://nomogram-xiangyahospital.shinyapps.io/BCLMOSnomogram) (accessed on 20 December 2022). (**D**) Web-based dynamic nomogram of CSS of BCLM patients (https://cssnomogram-xiangyahospital.shinyapps.io/DynNomapp) (accessed on 20 December 2022).

Based on risk stratification, Kaplan–Meier survival curves were plotted for all BCLM patients, as shown in Figure 5. The 5-year OS rate is the highest in the low-risk group at 34.5%, followed by the intermediate-risk group at 12.9%, and the worst at 4.9% in the high-risk group (Figure 5A). Likewise, the low-risk group had the highest 5-year CSS rate at 40.1%, followed by the intermediate-risk group with 14.5%, and the high-risk group with 4.6% (Figure 5B). There was a statistically significant difference in survival outcomes among the three groups (*p* < 0.001). We finally published our web-based nomogram for predicting OS in BCLM patients (Figure 5C) and CSS (Figure 5D), which can be accessed at (https://nomogram-xiangyahospital.shinyapps.io/BCLMOSnomogram) (accessed on 20 December 2022) and (https://cssnomogram-xiangyahospital.shinyapps.io/DynNomapp) (accessed on 20 December 2022) to conduct your data calculations and risk analysis. A step-by-step instruction was produced to help researchers use the site (Supplementary File).

## 4. Discussion

Risk assessment tools and surveillance methods for BCLM remain limited. We constructed a nomogram based on extensive population data from the SEER database and included breast cancer molecular subtypes, treatment modalities, and sociodemographic factors. Although Xie’s study found disease risk factors based on similar data, it did not build a complete risk assessment tool [12]. We developed and constructed a nomogram of an accurate scoring system with clinical value and achieved good classification results for OS and CSS in BCLM patients. We validated our model with multiple methods and found that it works well to provide comprehensive guidance for clinical practice. We published it online, so all readers can easily access and use it.

Our study found that surgical treatment is protective for OS and CSS in BCLM patients. Surgery can prolong the survival time of patients and reduce the risk of death by 29.1%. Several randomized clinical trials and retrospective studies have shown that primary tumor surgery can improve cancer survival by reducing the tumor burden of metastatic breast cancer [13,14,15]. However, selection bias cannot be ignored, such as selecting patients with smaller tumor burden, younger age, or other more favorable characteristics for surgery, which may lead to the final result being more biased towards the surgery group [16,17]. Some basic research suggests that surgical injury may upregulate genes involved in adhesion, invasion, and angiogenesis, leading to breast cancer metastasis in the lung [18]. Although the benefit of primary tumor surgery in patients with newly diagnosed breast cancer and lung metastases is unclear, our results suggest that BCLM patients undergoing surgical treatment have a survival benefit.

Our study found significant differences in the prognosis of BCLM patients of different molecular subtypes. We separately analyzed the three crucial therapeutic targets of breast cancer, ER, PR, and HER2, and the positive of the three reduced the risk of death by 48.9%, 44.2%, and 25.8%. This suggests that BCLM patients pay more attention to evaluating ER molecular targets. There is evidence of an interaction between the ER and HER2 pathways [19]. Zhao’s research also found that molecular typing strongly impacts metastatic breast cancer [20]. The ER pathway can be used as a bypass activation mechanism for downstream signals of the HER2 pathway [21]. Activating the HER2 signaling pathways could further promote the activity of the ER pathway, leading to impaired endocrine therapy response and possibly changing the tumor’s response to HER2-targeted therapy [22]. Some neoadjuvant clinical studies targeting anti-HER2 have shown that, compared with HER2+/ER- patients, HER2+/ER+ patients have a lower pathological complete response rate (pCR) [23]. It also suggests that our breast cancer patients with lung metastasis may be closely related to ER expression in molecular mechanisms.

In sociodemography, we found that marital status is an independent factor for BCLM patients. Marriage could reduce the risk of death by 25%, similar to our previous research on colorectal cancer [24]. However, more than 99% of breast cancer patients are women, and the hormone cycle and stable level are closely related to the occurrence and development of breast cancer [25]. Unmarried patients often lack the care and support of their spouses and are more likely to suffer from chronic psychological distress, such as depression and helplessness and unhealthy living habits, such as smoking and alcoholism [26]. Reducing psychosocial support and psychological stress will affect the immune and endocrine systems and accelerate tumor growth and patient death [27]. Furthermore, with the further development of the industrialization process, the human marriage rate may be further reduced [28]. Therefore, in clinical work, providing psychosocial support from medical workers may help improve the survival of patients.

Our study randomly selected one-third of the patients as the validation group to confirm the superiority of the nomogram in this study. The excellent results of the verification group, including the C index, time-dependent ROC curve, DCA, and calibration curve, ensure the generalizability of the new nomogram. We made our models as web-based dynamic nomograms, making our models available to everyone online. However, there are still some limitations in our research. First, as a retrospective study, the nomogram still needs to be verified by prospective studies in the future. Secondly, much information is missing in our research, such as the lack of metastatic tumor size, location, and treatment response, but with the further improvement of the database, these problems will be further solved. In addition, we need more accurate data to verify the validity of the nomogram. Despite these limitations, the findings demonstrate the clinical value of the sensitivity and specificity of our constructed nomogram.

## Figures and Tables

**Table 1:** Demographics and clinicopathologic characteristics of the cohort with breast cancer lung metastatic.

Variables	Total Cohort (*n* = 3916)		Training Cohort (*n* = 2611)		Validation Cohort (*n* = 1305)	
	No	%	No	%	No	%
Age						
≤50	788	20.1	516	19.8	272	20.8
51–65	1556	39.7	1045	40.0	511	39.2
>65	1572	40.1	1050	40.2	522	40.0
Gender						
Female	3853	98.4	2573	98.5	1280	98.1
Male	63	1.6	38	1.5	25	1.9
Marital status						
Married	1613	41.2	1073	41.1	540	41.4
Unmarried	2303	58.8	1538	58.9	765	58.6
Insurance						
No	228	5.8	146	5.6	82	6.3
Yes	3688	94.2	2465	94.4	1223	93.7
Race						
White	2830	72.3	1886	72.2	944	72.3
Black	729	18.6	469	18.0	260	20.0
Other	357	9.1	256	9.8	101	7.7
Grade						
I/II	1729	44.2	1149	44.0	580	44.4
III/IV	2187	55.8	1462	56.0	725	55.6
T stage						
T1	433	11.1	294	11.3	139	10.7
T2	1140	29.1	779	29.8	361	27.7
T3	691	17.6	452	17.3	239	18.3
T4	1652	42.2	1086	41.6	566	43.3
N stage						
N0	861	22.0	580	22.2	281	21.5
N1	1931	49.3	1279	49.0	652	50.0
N2	516	13.2	336	12.9	180	13.8
N3	608	15.5	416	15.9	192	14.7
Surgery						
No	2719	69.4	1807	69.2	912	69.9
Yes	1197	30.6	804	30.8	393	30.1
Chemotherapy						
No	1462	37.3	965	37.0	497	38.1
Yes	2454	62.7	1646	63.0	808	61.9
Radiation						
No	1689	43.1	754	28.9	935	71.6
Yes	2227	56.9	1857	71.1	370	28.4
Bone metastatic						
No	1829	46.7	1244	47.6	585	44.8
Yes	2087	53.3	1367	52.4	720	55.2
Brain metastatic						
No	3559	90.9	2370	90.8	1189	91.1
Yes	357	9.1	241	9.2	116	8.9
Liver metastatic						
No	2872	73.3	1921	73.6	951	72.9
Yes	1044	26.7	690	26.4	354	27.1
ER						
Negative	1194	30.5	803	30.8	391	30.0
Positive	2722	69.5	1808	69.2	914	70.0
PR						
Negative	1705	43.5	1132	43.4	573	43.9
Positive	2211	56.5	1479	56.6	732	56.1
HER2						
Negative	2770	70.7	1824	69.9	946	72.5
Positive	1146	29.3	787	30.1	359	27.5
OS (months)	20.94		20.9		20.9	
CSS (months)	21.01		21.0		20.9	

For marital status, unmarried consists of single, divorced, separated, and widowed. For race, ‘other’ includes American Indian, AK Native, Asian, and Pacific Islander. ER, estrogen receptor. PR, progesterone receptor. HER2, human epidermal growth factor receptor 2.

**Table 2:** Univariable and multivariable Cox regression model analyses of OS for nomogram.

Characteristics		Univariable Analysis			Multivariable Analysis	
	OR	95% CI	*p*	OR	95% CI	*p*
Age			<0.001			<0.001
≤50		Reference	1		Reference	1
51–65	1.132	0.976–1.312	0.101	1.143	0.984–1.328	0.081
>65	1.439	1.244–1.665	<0.001	1.517	1.297–1.774	<0.001
Gender			0.642			
Female		Reference	1			-
Male	0.902	0.597–1.362				
Marital status			<0.001			<0.001
Mariied		Reference	1		Reference	1
Unmarried	1.327	1.192–1.476	<0.001	1.254	1.124–1.399	<0.001
Insurance			0.21			
No		Reference	1			-
Yes	1.144	0.927–1.411				
Race			<0.001			<0.001
White		Reference	1		Reference	1
Black	1.317	1.157–1.499	<0.001	1.320	1.153–1.511	<0.001
Other	0.848	0.700–1.029	0.095	0.838	0.690–1.018	0.075
Grade			<0.001			0.008
I/II		Reference	1		Reference	1
III/IV	1.526	1.372–1.696	<0.001	1.480	1.311–1.670	<0.001
T stage			<0.001			<0.001
T1		Reference	1		Reference	1
T2	1.149	0.950–1.390	0.153	1.094	0.904–1.325	0.355
T3	1.343	1.093–1.649	<0.001	1.184	0.963–1.457	0.109
T4	1.486	1.239–1.782	<0.001	1.301	1.083–1.563	0.005
N stage			0.82			
N0		Reference	1			-
N1	1.00875743	0.884–1.152	0.897			
N2	0.951846599	0.794–1.141	0.593			
N3	1.044337376	0.883–1.235	0.612			
Surgery			<0.001			<0.001
No		Reference	1		Reference	1
Yes	0.711	0.635–0.796	<0.001	0.667	0.589–0.755	<0.001
Chemotherapy			<0.001			<0.001
No		Reference	1		Reference	1
Yes	0.811	0.730–0.901	<0.001	0.667	0.589–0.755	<0.001
Radiation			0.356			
No		Reference	1			-
Yes	0.949	0.849–1.061	0.356			
Bone metastatic			0.006			<0.001
No		Reference	1		Reference	1
Yes	1.156	1.042–1.282	0.006	1.257	1.122–1.409	<0.001
Brain metastatic			<0.001			<0.001
No		Reference	1		Reference	1
Yes	1.845	1.567–2.171	<0.001	1.753	1.482–2.072	<0.001
Liver metastatic			<0.001			<0.001
No		Reference	1		Reference	1
Yes	1.707	1.527–1.908	<0.001	1.694	1.507–1.905	<0.001
ER			<0.001			<0.001
Negative		Reference	1		Reference	1
Positive	0.527	0.473–0.587	<0.001	0.527	0.448–0.619	<0.001
PR			<0.001			<0.001
Negative		Reference	1		Reference	1
Positive	0.539	0.535–0.658	<0.001	0.756	0.647–0.882	<0.001
HER2			<0.001			<0.001
Negative		Reference	1		Reference	1
Positive	0.729	0.647–0.821	<0.001	0.608	0.535–0.692	<0.001

**Table 3:** Univariable and multivariable Cox regression model analyses of CSS for nomogram.

Characteristics		Univariable Analysis			Multivariable Analysis	
	OR	95% CI	*p*	OR	95% CI	*p*
Age			0.007			0.002
≤50		Reference	1		Reference	1
51–65	1.114	0.948–1.309	0.101	1.113	0.945–1.312	0.201
>65	1.285	1.092–1.513	<0.001	1.349	1.132–1.608	0.001
Gender			0.642			
Female		Reference	1			-
Male	0.902	0.597–1.362				
Marital status			<0.001			<0.001
Mariied		Reference	1	1.270	Reference	1
Unmarried	1.341	1.186–1.517	<0.001		1.12–1.441	<0.001
Insurance			0.21			
No		Reference	1			-
Yes	1.144	0.927–1.411				
Race			<0.001			<0.001
White		Reference	1	1.316	Reference	1
Black	1.369	1.183–1.583	<0.001	0.818	1.129–1.534	<0.001
Other	0.86	0.689–1.073	0.181		0.654–1.024	0.08
Grade			<0.001			<0.001
I/II		Reference	1	1.534	Reference	1
III/IV	1.569	1.388–1.774	<0.001		1.333–1.764	<0.001
T stage			<0.001			0.002
T1		Reference	1	1.219	Reference	1
T2	1.319	1.017–1.711	0.037	1.340	0.938–1.583	0.1390728
T3	1.617	1.231–2.123	0.001	1.507	1.018–1.764	0.0365868
T4	1.775	1.383–2.279	<0.001		1.172–1.938	0.0013814
N stage			0.82			
N0		Reference	1			-
N1	1.00875743	0.884–1.152	0.897			
N2	0.951846599	0.794–1.141	0.593			
N3	1.044337376	0.883–1.235	0.612			
Surgery			<0.001			<0.001
No		Reference	1	0.667	Reference	1
Yes	0.666	0.584–0.759	<0.001		0.590–0.777	<0.001
Chemotherapy			<0.001			<0.001
No		Reference	1	0.687	Reference	1
Yes	0.866	0.767–0.979	0.021		0.594–0.794	<0.001
Radiation			0.356			
No		Reference	1			-
Yes	0.949	0.849–1.061	0.356			
Bone metastatic			0.002			<0.001
No		Reference	1	1.325	Reference	1
Yes	1.21	1.072–1.363	0.002		1.161–1.511	<0.001
Brain metastatic			<0.001			<0.001
No		Reference	1	1.868	Reference	1
Yes	2.039	1.700–2.446	<0.001		1.549–2.253	<0.001
Liver metastatic			<0.001			<0.001
No		Reference	1	1.655	Reference	1
Yes	1.769	1.559–2.006	<0.001		1.449–1.891	<0.001
ER			<0.001			<0.001
Negative		Reference	1	0.550	Reference	1
Positive	0.511	0.452–0.578	<0.001		0.458–0.661	<0.001
PR			<0.001			<0.001
Negative		Reference	1	0.694	Reference	1
Positive	0.558	0.496–0.629	<0.001		0.580–0.830	<0.001
HER2			<0.001			<0.001
Negative		Reference	1	0.595	Reference	1
Positive	0.742	0.649–0.849	<0.001		0.514–0.688	<0.001

**Table 4:** The C-indices for predictions of OS and CSS.

Group	OS		CSS	
	C-Index	95% CI	C-Index	95% CI
Training group	0.702	0.687–0.716	0.710	0.693–0.727
Validation group	0.703	0.682–0.723	0.715	0.692–0.737

OS, overall survival; CSS, cancer-specific survival; C-index, index of concordance; CI, confidence interval.

## Data Availability

All patient files are available from the SEER database. More details supporting the findings of this study are available from the corresponding author upon request.

## Supplementary Materials

The following are available online at https://www.mdpi.com/article/10.3390/jpm13010043/s1, File: Web-based dynamic nomograms for predicting overall survival and cancer-specific survival in breast cancer patients with lung metastases.
