# Supplementary material for: Web-Based Dynamic Nomograms for Predicting Overall Survival and Cancer-Specific Survival in Breast Cancer Patients with Lung Metastases
Source: J Pers Med. 2022 Dec 26;13(1):43. doi: 10.3390/jpm13010043 (PMC9864097; doi:10.3390/jpm13010043)
Supplement: Supplementary file 1 [file jpm-13-00043-s001.zip › jpm-2010953-supplementary.pdf]

Supplement

Web-based dynamic nomograms for predicting overall survival and cancer-specific survival in breast cancer patients with lung metastases

Kangtao Wang, Yuqiang Li, Dan Wang, Yuan Zhou and Zhongyi Zhou

Table of Content

Supplemental information 1 Step-by-step instruction for web-based dynamic nomograms

Supplemental information 1 Step by step instruction

1. Obtain the general situation of the patient: Age, Marital status, Race
2. Obtain the status of the patient's tumor: Grade, T-stage, Surgery, Chemotherapy
3. Obtain patient metastasis: Bone metastasis, Brain metastasis, Liver metastasis
4. Obtain the patient's tumor pathology: ER, PR, HER2
5. Open the predicting OS in BCLM patients (https://nomogram-xiangyahospital.shinyapps.io/BCLMOSnomogram) or CSS (https://cssnomogram-xiangyahospital.shinyapps.io/DynNomapp) in your browse.

Here we take an example of a 55-year-old, married, white, Grade III, T3 patient with TNBC who has undergone surgery, chemotherapy, bone metastases, no liver, brain metastases, and lung metastases.

We want to predict the patient's 6-month survival rate. Then we can speculate, based on the nomogram, that the probability of the patient surviving for more than two months is only more than 25%, as Figure S1 shown. The estimated survival time was only 0.032 months (95% CI). As Figure S2 shown. You can view the specific data in the Numerical Summary.

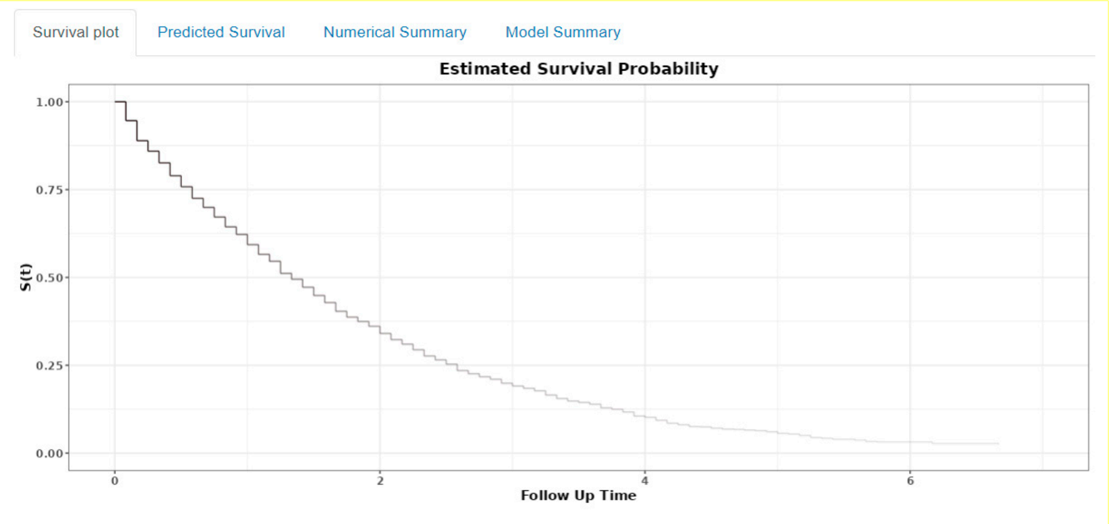

Figure S1. Example Patient Prediction K-M Plot.

Survival plot | Predicted Survival | Numerical Summary | Model Summary

ge	Surgery	Chemotherapy	Bonemetastasis	Brainmetastasis	Livermetastasis	ER	PR	HER2	Prediction	Lower.bound	Upper.bound
	Yes	Yes	Yes	Yes	No	Negative	Negative	Negative	0.032	0.015	0.070

Figure S2.Example Patient Numerical Summary data.

6. If you cannot get some patient information, we have found the model to

be still instructive. For example, the pathological results of the patient may not be obtained yet, and it is impossible to know the results of the patient's ER, PR, and HER2. We still take the above patient as an example. We select ER, PR, and HER2 as positive(Figure S3). After the input, it was found that the patient's survival probability had greatly improved. Then the predicted patient probability should be within this interval, and the method used is between the best and worst estimates.

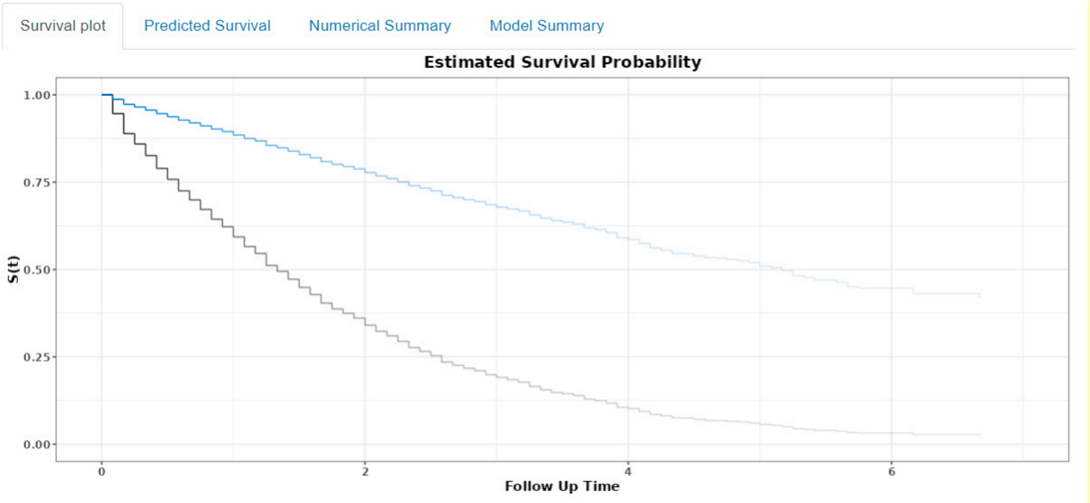

Figure S3. Predictive K-M plot for patients with incomplete information.

Survival plot | Predicted Survival | Numerical Summary | Model Summary

ge	Surgery	Chemotherapy	Bonemetastasis	Brainmetastasis	Livermetastasis	ER	PR	HER2	Prediction	Lower.bound	Upper.bound
	Yes	Yes	Yes	Yes	No	Negative	Negative	Negative	0.032	0.015	0.070
	Yes	Yes	Yes	Yes	No	Positive	Positive	Positive	0.450	0.360	0.550

Figure S4. Patient with incomplete information Numerical Summary data.
